# Sex-related differences in parental rearing patterns in young adults with bipolar disorder

**DOI:** 10.1038/s41598-023-48576-6

**Published:** 2023-12-08

**Authors:** Huifang Zhao, Xujing Zhang, Meihong Xiu, Fengchun Wu

**Affiliations:** 1 Hebei Province Veterans Hospital, Baoding, China; 2 Hebei Province Mental Health Center, Baoding, China; 3 Peking University HuiLongGuan Clinical Medical School, Beijing HuiLongGuan Hospital, Beijing, China; 4 Department of Psychiatry, The Affiliated Brain Hospital of Guangzhou Medical University, Guangzhou, China; 5 Department of Biomedical Engineering, Guangzhou Medical University, Guangzhou, China; 6 Guangdong Engineering Technology Research Center for Translational Medicine of Mental Disorders, Guangzhou, China

**Keywords:** Schizophrenia, Psychiatric disorders

## Abstract

The aim of this study was to examine the parenting characteristics of young patients with bipolar disorder (BD) and explore the sex differences. The parental rearing pattern of young patients with BD was measured and compared with the healthy control of young adults. The EMBU scale was used to assess parental rearing patterns. Patients with BD reported significantly higher scores in the punishment and severity index, as well as of the rejection and denial index, but lower scores in the warmth & affectionate index in the paternal rearing pattern, compared with healthy controls. In addition, patients scored higher on the punishment and severity index and rejection and patterns index in maternal rearing patterns. More importantly, we found significant sex differences in maternal rearing patterns (p_Bonferroni_ < 0.05). Specifically, in the maternal rearing patterns, male patients had higher scores on the favoring index than male controls, whereas female patients had lower scores on the warmth & affectionate index than female controls. This study shows significant differences in parental rearing patterns between patients and control subjects. Male patients were overprotective by their mothers and female patients were overlooked by their mothers during upbringing.

## Introduction

Bipolar disorder (BD) is a chronic disease characterized by fluctuations in mood state and energy. The lifetime prevalence is estimated to be approximately 1–2%, irrespective of nationality, ethnic origin or socioeconomic status^1^. Patients with BD have diverse clinical manifestations and a high rate of suicide, with approximately 1/3 admitting to at least one suicide attempt^2^. The usual onset age of BD is before the age of 30 years^3^, and BD is one of the leading causes of disability in young people^4^. However, the etiology of BD is not fully understood. Recent studies in patients with BD have suggested an interaction between genetic and environmental factors in the pathogenesis of the disease^5–7^, particularly parental rearing patterns and familial factors among the environmental factors.

Parent–child attachment relationship is a two-way process in which children develop emotional attachments to their caregivers^8,9^. Parental rearing patterns play a crucial role in the mental health of adolescents^10^. Good family and parent–child relationships provide the necessary environment for their development, contribute to child health and constitute overall public health^11,12^. Negative parenting styles such as refusal, denial, and rejection have a negative impact on children's mental health and are risk factors for psychological disorders^13–17^. In contrast, positive and supportive parenting styles such as warmth, responsiveness, and affirmative play a positive role in children's personality traits, social interactions, and self-evaluation^18^. All evidence suggests a critical role of parent rearing patterns in the development of certain common psychiatric disorders.

Indeed, there is growing evidence of differential associations between parental attitudes and behaviors in daily interactions with their children, characterized by parental rearing patterns that are associated with a variety of mental diseases^19–23^. For example, in a previous prospective adoption study, it was reported that parenting patterns interacted with genetic underpinnings to increase the risk of schizophrenia^24^. In another study, lower baseline parental care was strongly associated with the development of psychosis^25^. Studies have shown that parental rearing patterns are not only related to age of onset, but also to the illness course or outcome of mental disorders^26,27^. Regarding patients with BD, most previous studies have found negative parenting styles, such as neglectful parenting and inadequate protection and care^28,29^.

Sex differences in onset, certain symptom profiles, and disease outcomes in patients with BD have been widely reported^30–33^. While most, but not all, studies have shown no gender differences in the prevalence of bipolar disorder, the majority of studies do show that women are at higher risk of developing BD characterized by rapid cycling and mixed episodes^34^. Sex differences have also been found in co-morbidities^35^. Notably, the existing literature indicates that sex differences also exist in parental rearing patterns. For example, there is a significant interaction effect of sex and parental rearing pattern on the levels of delinquency behaviour. In addition, higher rates of delinquency have been associated with neglectful parenting among males and permissive parenting among females^36^. Parental rearing patterns have been reported to have differential effects on the development of cyber aggression in children, depending on the sex of the offspring^37^. A previous meta-analytic review also reported that the relationships between maternal and paternal rearing patterns and relational aggression among adolescents depended on the child’s gender^38^. However, no studies have been conducted to investigate whether young patients with BD exhibit sex differences in parenting rearing patterns. The main purpose of the current study was to address whether patients with BD differ from healthy controls in their parental rearing patterns. Furthermore, we explored sex-based differences in parenting styles.

## Methods

### Subjects

From April 2012 to May 2013, a total of 40 patients with BD were recruited from Hebei Mental Health Center. Inclusion criteria included: (1) bipolar I disorder diagnosed according to Diagnostic and Statistical Manual of Mental Disorders, 4th Edition (DISM-IV) criteria; (2) 16 years of age or above; (3) educational level: elementary school or above; (4) being able to cooperate to complete the questionnaire, and (5) consent to sign an informed consent form. Exclusion criteria included: (1) other mental disorders diagnosed by DSM-IV; (2) having any comorbid severe physical illness or organic brain disease; (3) past or current substance abuse/dependence (excluding alcohol and cigarette); and (4) mental retardation.

Additionally, a total of 40 age- and sex-matched healthy controls (HC) were recruited from the local community. Medical history, physical examination, laboratory tests, current mental status and family history of any mental illness were evaluated by a psychiatrist. HC subjects were in good physical health and any subjects with chronic physical diseases such as diabetes, coronary heart disease, hypertension, drug and alcohol abuse dependence intellectual disability or other organic brain diseases were rigorously excluded. In addition, HC subjects with a current or past history of psychiatric disorders were also excluded through structured SCID interviews.

All research and data collection processes were conducted in accordance with the Declaration of Helsinki and current ethical guidelines. The procedures and protocol were reviewed and approved by an independent ethics committee of Hebei Mental Health Center. All participants were informed of the study orally and in writing before the start of the study. Informed consent was obtained from all participants or their next of kin.

### Parents rearing behavior evaluation

Perceived parental rearing patterns were assessed by the EMBU scale (Egna Minnen av Barndoms Uppfostran) developed by Perris et al.^39,40^. It was previously translated into Chinese by Dr. Yue and its clinical validity and test–retest reliability were established^41,42^. The EMBU consists of 55 subtests used to calculate 6 index scores for paternal rearing patterns (warmth & affectionate, punishment and severe, favoring, rejection, over-intervention and overprotection) and 5 index scores for maternal rearing patterns (warmth & affectionate, punishment and severe, favoring, rejection, over-intervening, and overprotection). Prior to the start of this study, the psychiatrists were trained in the use of the EMBU scale. After training, repeated assessments showed that the inter-rater correlation coefficient for each index score remained above 0.90. All recruited patients and HC subjects completed the EMBU scale. For patients, the EMBU scale was assessed when patients had a Bech-Rafaelsen Mania Scale (BRMS) score of < 5 and a Hamilton Depression Rating Scale (HDRS) score of < 8 (i.e., remission).

### Statistical analysis

Sample Size Power was calculated based on expected changes in parental rearing patterns. The sample size is considered to achieve significance with a moderate effect size (ES) (d = 0.30), a power of 80%, and α = 0.05. The Shapiro–Wilk’s test was performed to evaluate whether EMBU scores were in a normal distribution across all participants. Demographic variables were compared between patients and HCs by using chi-square for categorical variables or t-test for normally distributed continuous variables.

Then, a two-way analysis of variance (ANOVA) was performed to investigate the differences in parental rearing patterns between patients and HCs. We focused more on the interaction effect of diagnostic group (patients vs controls) and sex (male vs female) on parental rearing behaviors.

Statistical analyses were performed using SPSS version 20.0. The significance level for differences was set at < 0.05. Bonferroni correction was used for multiple comparisons.

## Results

### Clinical and demographic data

The demographic and clinical characteristics of the patient and HC groups are shown in Table 1. There were no significant differences in terms of age, sex, race, or parental educational level between patients and controls (all *p* > 0.05). However, significant differences were found in educational level and the number of cigarettes smoked by the participants (all *p* < 0.05).

**Table 1:** Demographic and clinical characteristics of patients and control subjects by using t-tests and chi-square.

Characteristic	Patients (*n* = 40)	Controls (*n* = 40)	t (p)
Demographic characteristics			
Age, mean ± SD, y	31.7 ± 9.3	28.6 ± 8.0	1.6 (0.12)
Educational level, mean ± SD, y	9.6 ± 2.7	12.6 ± 3.4	4.6 (< 0.001)
Male sex	24/16	24/16	< 0.001 (1.0)
Marital status			0.66
Currently married	21	19	
Single or divorced	19	21	
Race			0.2 (0.64)
Han	38	37	
Mongolian	2	3	
Nonsmokers/smokers, n/n	26/12	28/9	0.5 (0.48)
Years of smoking, mean ± SD	13.3 ± 11.0	12.1 ± 5.8	0.3 (0.78)
Nondrinking/drinking, n/n	29/9	28/9	0.004 (0.95)
Number of drinks per day	3.8 ± 3.3	1.3 ± 0.5	2.1 (0.08)
Years of drinks, mean ± SD, y	9.4 ± 9.5	14.1 ± 7.0	1.1 (0.27)
Clinical characteristics			
Type of BD			
Bipolar I	40 (100%)		
Onset age, mean (SD), y	24.7 ± 8.4		
Duration of illness, mean ± SD, y	7.4 ± 7.0		
Number of episodes	3.4 ± 2.7		
Number of hospitalizations	2.3 ± 1.3		
Family characteristics			
Number of family living together	3.8 ± 1.7	3.1 ± 1.2	1.9 (0.06)
Live with both parents until, mean ± SD, y	23.5 ± 5.3	21.6 ± 3.6	1.8 (0.07)
Father deceased (No)	34/5	33/5	0.002 (0.97)
Mother deceased (No)	34/6	38/0	6.2 (0.03)
Parent divorced (No)	40/0	37/1	1.1 (0.49)
Educational level of father, mean ± SD, y	8.0 ± 2.0	9.3 ± 3.6	2.0 (0.054)
Educational level of mother, mean ± SD, y	7.3 ± 2.9	7.7 ± 3.2	0.6 (0.53)

In addition, we found significant associations between sex and paternal rearing patterns, including punishment and over-interference index and rejection and denial index in the entire group, or when analyzing patients and controls separately. However, in both the patient and control groups, we did not find a significant association between sex and maternal rearing patterns (Table 2).

**Table 2:** Comparison of parental rearing patterns (mean ± SD) by using t-tests.

EMBU subscores	Patients (*n* = 40)	Controls (*n* = 40)	*t*	*P*
Paternal rearing patterns				
Warmth & affectionate	29.0 ± 11.3	33.1 ± 6.0	4.0	0.05*
Punishment and severe	8.2 ± 5.0	3.6 ± 3.1	23.9	< 0.001**
Overinterference	10.5 ± 3.8	10.3 ± 3.7	0.03	0.86
Favoring	6.1 ± 3.4	4.7 ± 3.1	3.4	0.07
Rejection and denial	4.6 ± 3.1	2.2 ± 1.7	17.8	< 0.001**
Overprotection	5.8 ± 2.6	5.3 ± 2.9	0.7	0.20
Maternal rearing patterns				
Warmth & affectionate	31.5 ± 11.0	34.3 ± 7.9	1.6	0.20
Overinterference and overprotection	15.8 ± 6.3	17.5 ± 6.8	5.3	0.024*
Rejection and denial	6.8 ± 4.1	3.8 ± 3.52	12.4	0.001**
Punishment and severe	5.4 ± 4.4	2.4 ± 2.8	13.7	< 0.001**
Favoring	6.4 ± 3.5	5.1 ± 3.5	2.6	0.11

*p < 0.05; **p < 0.01.

### Parental rearing behaviors in patients and controls

For the paternal rearing patterns, we found that patients with BD scored higher than HC subjects on the punishment and severe index, as well as in the rejection and denial index, but lower on the emotional warmth & affectionate index. P values for all these differences passed the strict Bonferroni correction (all p_Bonferroni_ < 0.01) (Table 2).

For the maternal rearing behaviors, patients with BD scored higher on punishment and severe index, rejection and denial index, as well as over-interference and overprotection index (all *p* < 0.05). After Bonferroni correction, the differences in punishment and severe index, as well as rejection and denial index between patients and controls remained significant (all p_Bonferroni_ < 0.05) (Table 2).

### Sex differences in parental rearing behaviors

We then analyzed sex differences in parenting behaviors between patients and controls. There was a significant interaction between sex and group on the warmth & affectionate index and the favoring index for paternal rearing pattern, as well as emotional warmth & affectionate and favoring index (all *p* < 0.05) (Tables 3, 4). On the warmth & affectionate dimension of paternal parenting styles, male patients scored higher than female patients, while male controls scored lower than female controls. On the favoring dimension of paternal parenting styles, male patients had higher scores than female patients, whereas no difference was observed in HC subjects. However, all these differences did not pass the strict Bonferroni corrections (all p_Bonferroni_ > 0.05).

**Table 3:** Sex difference in parental rearing patterns in the entire group including patients and controls (mean ± SD) by using t-tests.

EMBU subscores	Males (*n* = 48)	Females (*n* = 32)	*t*	*P*
Maternal rearing patterns				
Warmth & affectionate	32.8 ± 8.7	32.8 ± 11.2	0.001	0.98
Over-interference and overprotection	18.5 ± 7.1	16.1 ± 6.1	2.5	0.12
Rejection and denial	5.8 ± 4.3	4.5 ± 3.8	2.0	0.17
Punishment and severe	4.2 ± 4.2	3.3 ± 3.5	0.99	0.32
Favoring	5.8 ± 3.6	5.6 ± 3.6	0.08	0.78
Paternal rearing patterns				
Warmth & affectionate	31.1 ± 9.1	30.8 ± 9.6	0.02	0.90
Punishment and severe	7.1 ± 5.0	4.3 ± 4.0	6.7	0.01*
Excessive interference	11.2 ± 4.1	9.1 ± 2.9	6.7	0.01*
Favoring	5.2 ± 3.2	5.8 ± 3.6	0.7	0.42
Rejection and denial	4.0 ± 2.9	2.4 ± 2.0	7.4	0.008**
Overprotection	6.1 ± 2.9	4.8 ± 2.2	3.9	0.052

*p < 0.05; **p < 0.01.

**Table 4:** Sex difference in parental rearing patterns in patients and controls (mean ± SD).

	Patients		Controls		Effect		
	Males	Females	Males	Females	Group F (p)	Sex F (p)	Interaction F (p)
Paternal rearing patterns							
F1	31.4 ± 11.7	26.4 ± 9.1	31.2 ± 5.3	36.1 ± 5.9	5.0 (0.028)*	0.001 (0.98)	5.6 (0.02)*
F2	9.4 ± 5.1	6.5 ± 4.6	4.4 ± 3.2	2.2 ± 2.0	22.8 (0.017)*	7.0 (0.01)*	0.1 (0.72)
F3	11.2 ± 4.1	9.6 ± 3.1	10.7 ± 4.1	9.0 ± 2.6	0.4 (0.54)	3.7 (0.06)	0.005 (0.94)
F4	6.6 ± 3.5	5.4 ± 3.4	6.3 ± 3.9	6.3 ± 3.9	1.6 (0.21)	0.7 (0.41)	5.6 (0.02)*
F5	5.5 ± 3.1	3.1 ± 2.4	2.4 ± 1.9	1.9 ± 1.4	14.5 (< 0.001)**	6.7 (0.01)	3.0 (0.09)
F6	6.6 ± 2.8	4.7 ± 1.9	5.6 ± 3.4	5.0 ± 2.8	0.2 (0.65)	3.6 (0.06)	0.9 (0.35)
Maternal rearing patterns							
M1	35.0 ± 8.4	27.3 ± 12.6	33.0 ± 7.4	38.8 ± 4.9	5.1 (0.03)*	0.2 (0.65)	10.3 (0.002)**
M2	20.6 ± 7.3	18.1 ± 5.3	17.0 ± 5.9	14.5 ± 6.4	5.6 (0.02)*	2.7 (0.11)	0.00 (0.99)
M3	7.1 ± 3.9	6.2 ± 4.5	3.4 ± 2.6	3.0 ± 1.9	17.0 (< 0.001)**	0.6 (0.43)	0.09 (0.77)
M4	5.7 ± 4.7	5.0 ± 4.0	2.4 ± 2.4	1.7 ± 1.7	14.6 (< 0.001)**	0.7 (0.42)	0.003 (0.96)
M5	7.2 ± 3.3	4.9 ± 3.5	3.3 ± 1.8	6.3 ± 3.8	2.6 (0.11)	0.2 (0.66)	11.8 (0.001)**

F1 Warmth & affectionate; F2 Punishment and severe; F3 Over-interference; F4 Favoring; F5 Rejection and denial; F6 Overprotection.

M1 Warmth & affectionate; M2 Over-interference and overprotection; M3 Rejection and denial; M4 Punishment and severe, and M5 Favoring.

*p < 0.05; **p < 0.01.

**Figure 1:**
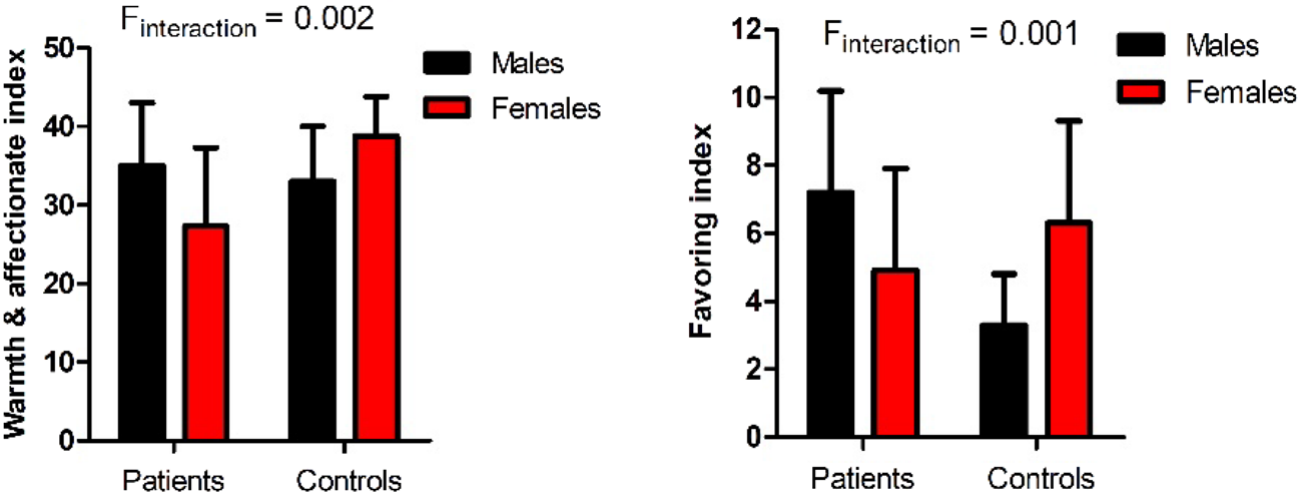
There were significant interaction effect of group and sex on the Warmth & affectionate index and Favoring index of maternal rearing patterns.

On the warmth & affectionate dimension of maternal parenting styles, male patients scored higher than female patients, while male controls scored lower than female controls. On the favoring dimension of the maternal rearing pattern, male patients had higher scores than female patients, whereas male controls scored lower than female controls (all *p* < 0.05). In addition, male patients scored higher on the favoring dimension than male controls (F = 11.4, *p* = 0.002), while female patients reported lower scores on the warmth & affectionate dimension than female controls (F = 11.0, *p* = 0.002). After Bonferroni correction, the difference remained significant (all p_Bonferroni_ < 0.05) (Fig. 1).

## Discussion

The present study demonstrated that (1) young patients with BD scored higher on punishment, harshness, rejection, and denial index assessed by the EMBU scale. (2) Sex differences were observed in patients. Males reported higher scores than females on the rejection and denial index. (3) The interaction between sex and diagnostic group was significant for the warm & affectionate index and favoring index of maternal parenting styles.

Compared to HC subjects, we found that young patients with BD scored higher on punishment, rejection, and denial index of parental rearing patterns, which is consistent with other studies in adolescents with BD^43,44^. It is noteworthy that patients and controls were well matched in terms of demographic data, personality, and family characteristics in the present study. Indeed, previous studies have provided strong evidence that parental rearing patterns are correlated with disease onset, disease course and outcomes of the disorder^23,45,46^. Parenting style is a fundamental component of child development and growth, as well as the background for many aspects of people’s personality, psychology, attitudes, feelings, and habits^47^. More importantly, the links between negative parental rearing patterns and the onset of BD later have been reported in several studies from divergent cultural backgrounds. Heider et al. found that significant correlations between parental rearing patterns and mood disorders were mostly homogeneous across six countries^22^. Negative parental rearing practices can have lasting and detrimental impacts on a person’s physical and mental health^48^. On the other hand, poor parenting styles increase potentially negative psychological traits in early adulthood, which can reduce well-being^49^. Among adults with severe mood disorders, cumulative exposure to adverse childhood experiences, including physical abuse, and sexual abuse, contributes to poorer mental health and worse functional outcomes^50,51^. Although twin studies have highlighted the fundamental role of genetic factors in the etiology of BD, the complex interplay between genetic and childhood adversity has been hypothesized to be the ultimate cause of BD pathogenesis^52^. For example, a study of adoptive and nonadoptive children reported socioeconomic disadvantages in childhood increased the risk in those with genetic liability for psychosis^53^. Thus, parental rearing styles are also critical and may be a predictive marker for the development of certain mental diseases later in life. Positive parental rearing patterns in childhood may have a positive effect on the risk of developing BD.

We further found that there were sex-specific differences in the parenting styles in young patients with BD. Our study demonstrates for the first time that there were significant differences in maternal rearing styles between male and female young patients with BD. More specifically, male patients were more likely than female patients to report higher levels of paternal caring, including emotional warmth and favoring. Conversely, male controls reported lower levels of paternal caring than female controls. Nevertheless, since the Bonferroni-corrected difference was not significant, it was considered a weak effect. Notably, on the dimension of emotional warmth in maternal parenting styles, male patients reported higher levels than female patients, but not controls. Moreover, male patients reported higher scores on the favoring index compared with male controls, whereas female patients reported lower scores on the warmth & affectionate index than female controls. Also, these differences remained significant after the Bonferroni correction. Our findings are consistent with a recent nationally representative cross-sectional survey of 6483 adolescents^54^, which found that girls reported higher levels of paternal control and lower levels of paternal care than boys. It should be noted that this study differs markedly from ours. Eun et al. reported sex differences in paternal parenting styles among adolescents with a variety of mental disorders, whereas our study found sex differences in maternal parenting styles only among young patients with BD.

However, we did not know the exact reason why the only significant sex difference was in the maternal parenting style rather than in the paternal parenting style. One possible explanation is that in China, especially in rural areas, children are mainly cared for by their mothers, who spend most of their time caring for them. In contrast, their fathers spend most of their time earning money to support the family. In addition, due to socioeconomic and cultural causes, some parents and grandparents are particularly patriarchal and spoiled boys. As a result, mothers have a greater influence on their children. On the other hand, girls receive much less attention from their mothers. Our findings suggest the vast differences in parenting styles between boys and girls, as well as between BD patients and controls.

Several limitations of this study should be noted. First, we did not investigate the potential effects of other risk factors, such as childhood adversity, substance use, and personality pathology. Second, we cannot make a conclusion that parental rearing patterns were the ultimate cause of the onset of BD, because the assessment was conducted during BD. Additionally, there may be a bidirectional relationship between the issues related to parenting and diagnosis. While it is possible that certain parenting styles may contribute to the development of BD, it is also possible that children with BD already exhibit some early behavioral issues that may affect the way they were raised by their parents, including the current state of the disorder, psychosocial function and global functioning. In particular, families caring for BD patients with severe dysfunction might have poorer family functioning. Therefore, there is an association here, but no cause-and-effect relationship can be established. Third, the small sample size of the current study is a methodological limitation that may lead to false positive or negative results due to a lack of statistical power. However, the gender frequencies of the patient and control groups were well matched (male: 24 vs female: 16 in both groups). Fourth, the relationship between parental rearing patterns and the pathogenesis of BD is not as appropriate as the hypothesis of this study. The impact of the existing disorder on the relationship cannot be excluded. A longitudinal design is required for this hypothesis. Fifth, it should be noted that the data in this study were collected more than 10 years ago. While our findings remain intriguing, it is not clear whether historical changes in awareness and raising styles may have affected the results.

In summary, the present study found significantly higher levels in the dimensions of punishment, denial and rejection of parenting styles in young patients with BD compared to healthy controls. Our findings suggest that parental rearing patterns may be associated with the risk of developing BD in young populations. In addition, significant sex differences were observed in the parenting styles of patients with BD. Male patients scored higher on maternal caring than male controls, indicating an overprotective parenting style during childhood. Therefore, parents should not adopt negative parenting styles such as punishment, rejection, and denial. In particular, mothers should not over-indulge their boys to facilitate personality development and psychological well-being and to reduce the occurrence of BD.

### Supplementary Information

Supplementary Information.

## Data Availability

The datasets used and/or analysed during the current study available from the corresponding author on reasonable request.
